# Evaluation of Human Hepatocyte Drug Metabolism Carrying High-Risk or Protection-Associated Liver Disease Genetic Variants

**DOI:** 10.3390/ijms241713406

**Published:** 2023-08-29

**Authors:** Lanuza A. P. Faccioli, Zeliha Cetin, Zehra N. Kocas-Kilicarslan, Kimberly Ortiz, Yiyue Sun, Zhiping Hu, Takeshi Kurihara, Edgar N. Tafaleng, Rodrigo M. Florentino, Zi Wang, Mengying Xia, Mark T. Miedel, D. Lansing Taylor, Jaideep Behari, Alina Ostrowska, Robert Constantine, Albert Li, Alejandro Soto-Gutierrez

**Affiliations:** 1 Department of Pathology, University of Pittsburgh School of Medicine, Pittsburgh, PA 15261, USA; cetinz@pitt.edu (Z.C.); zehranur.kocas@gmail.com (Z.N.K.-K.); ortiz.kimberly@medstudent.pitt.edu (K.O.); sunyiyue@pitt.edu (Y.S.); zhh69@pitt.edu (Z.H.); tak214@pitt.edu (T.K.); ent8@pitt.edu (E.N.T.); rodrigomachado@pitt.edu (R.M.F.); alina.ostrowska@chp.edu (A.O.); 2 Pittsburgh Liver Research Center, Human Synthetic Liver Biology Core, University of Pittsburgh, Pittsburgh, PA 15261, USA; dltaylor@pitt.edu (D.L.T.); behajx@upmc.edu (J.B.); 3 Department of Statistics, University of Pittsburgh, Pittsburgh, PA 15213, USA; ziw43@pitt.edu; 4 Drug Discovery Institute, University of Pittsburgh, Pittsburgh, PA 15261, USA; summer.xia@pitt.edu (M.X.); mmiedel@pitt.edu (M.T.M.); 5 Department of Computational and Systems Biology, University of Pittsburgh, Pittsburgh, PA 15260, USA; 6 Department of Medicine, Division of Gastroenterology, Hepatology, and Nutrition, University of Pittsburgh Medical Center, Pittsburgh, PA 15213, USA; 7 Discovery Life Sciences, Huntsville, AL 35806, USA; robert.constantine@dls.com (R.C.); albert.li@dls.com (A.L.); 8 McGowan Institute for Regenerative Medicine, Pittsburgh, PA 15219, USA

**Keywords:** metabolic-dysfunction-associated steatotic liver disease, metabolic dysfunction-associated steatohepatitis, nonalcoholic steatohepatitis, end-stage liver disease, genetic polymorphisms

## Abstract

Metabolic-dysfunction-associated steatotic liver disease (MASLD), which affects 30 million people in the US and is anticipated to reach over 100 million by 2030, places a significant financial strain on the healthcare system. There is presently no FDA-approved treatment for MASLD despite its public health significance and financial burden. Understanding the connection between point mutations, liver enzymes, and MASLD is important for comprehending drug toxicity in healthy or diseased individuals. Multiple genetic variations have been linked to MASLD susceptibility through genome-wide association studies (GWAS), either increasing MASLD risk or protecting against it, such as *PNPLA3* rs738409, *MBOAT7* rs641738, *GCKR* rs780094, *HSD17B13* rs72613567, and *MTARC1* rs2642438. As the impact of genetic variants on the levels of drug-metabolizing cytochrome P450 (CYP) enzymes in human hepatocytes has not been thoroughly investigated, this study aims to describe the analysis of metabolic functions for selected phase I and phase II liver enzymes in human hepatocytes. For this purpose, fresh isolated primary hepatocytes were obtained from healthy liver donors (n = 126), and liquid chromatography–mass spectrometry (LC–MS) was performed. For the cohorts, participants were classified into minor homozygotes and nonminor homozygotes (major homozygotes + heterozygotes) for five gene polymorphisms. For phase I liver enzymes, we found a significant difference in the activity of CYP1A2 in human hepatocytes carrying *MBOAT7* (*p =* 0.011) and of CYP2C8 in human hepatocytes carrying *PNPLA3* (*p* = 0.004). It was also observed that the activity of CYP2C9 was significantly lower in human hepatocytes carrying *HSD17B13* (*p* = 0.001) minor homozygous compared to nonminor homozygous. No significant difference in activity of CYP2E1, CYP2C8, CYP2D6, CYP2E1, CYP3A4, ECOD, FMO, MAO, AO, and CES2 and in any of the phase II liver enzymes between human hepatocytes carrying genetic variants for *PNPLA3* rs738409, *MBOAT7* rs641738, GCKR rs780094, *HSD17B13* rs72613567, and *MTARC1* rs2642438 were observed. These findings offer a preliminary assessment of the influence of genetic variations on drug-metabolizing cytochrome P450 (CYP) enzymes in healthy human hepatocytes, which may be useful for future drug discovery investigations.

## 1. Introduction

Nonalcoholic fatty liver disease (NAFLD), or, as has recently been proposed, metabolic-dysfunction-associated steatotic liver disease (MASLD), is a huge financial burden on the healthcare system. It affects 30 million adults in the US and is expected to increase to over 100 million by 2030 [1]. MASLD encompasses a spectrum of liver damage ranging from simple steatosis (or NAFL) to metabolic-dysfunction-associated steatohepatitis (MASH), cirrhosis, end-stage liver disease (ESLD), and hepatocellular carcinoma (HCC). Genetic and environmental factors, as well as disease drivers such as inflammatory cytokines, including adipokines, bacterial products, and metabolites originating from the intestine and adipose tissue, contribute to the development and progression of MASLD [2]. The pathologic hallmarks of MASLD include steatosis, inflammatory infiltrate, hepatocyte ballooning, and fibrosis, leading to decreased hepatocellular functions and eventually cirrhosis and HCC [3,4]. Despite its public health importance and financial burden, there is currently no FDA-approved therapy for MASLD. The lack of therapeutic options reflects the complex interpatient pathogenesis and heterogeneity as well as the lack of experimental models that fully recapitulate disease phenotypes and genotypes involved in MASLD progression.

Genome-wide association studies (GWAS) have identified several genetic variants that are associated with MASLD susceptibility, including single-nucleotide polymorphisms (SNP) in *PNPLA3* (rs738409 C>G p.Ie148Met; patatin-like phospholipase-domain-containing protein 3), *MBOAT7* (rs62641738 C>T; membrane-bound O-acyltransferase-domain-containing 7), and *GCKR* (rs780094 C>T; glucokinase regulator) [5].

The most relevant and reproducible SNP identified across GWAS is in the patatin-like phospholipase-domain-containing 3 (*PNPLA3*) gene (rs738409 C>G p.IIe148Met) [6]. This variant encodes an isoleucine-to-methionine substitution at position 148aa (I148M) in the protein, which causes PNPLA3’s lipase activity to be lost, resulting in fat buildup in hepatocytes. A decrease in protein function is also caused by the MBOAT7 rs62641738 genetic variation, which causes it to lose its ability to acetylate lysophosphatidylinositol lipids and, therefore, its protective role in preventing hepatic steatosis [7,8]. GWAS have revealed a significant link between GCKR genetic variation and various metabolic parameters. Specifically, the p.P446L GKRP substitution has been identified as a critical factor; this substitution leads to the destabilization of the GCK binding interface, explaining the observed inverse correlation between fasting glucose and triglycerides for individuals carrying this variant. Furthermore, the increased activity of hepatic GCK resulting from this genetic variation leads to reduced glucose levels and simultaneous increases in triacylglycerol (TAG) and glycogen synthesis in individuals with normal blood sugar levels (normoglycemia) [9].

In contrast to variants that increase MASLD risk, recent studies have identified two novel protective variants in the *HSD17B13* (rs72613567 T>TA; 17-β hydroxysteroid dehydrogenase 13) and *MTARC1* (rs2642438 G>A p.Ala165Thr; mitochondrial amidoxime reducing component 1) genes that are linked to lower risk of MASLD [10,11,12,13,14]. However, little is currently known regarding the biological function of these MASLD high-risk or protective variants. *HSD17B13* is a lipid-droplet-associated protein with retinol dehydrogenase activity. Hepatic expression of *HSD17B13* has been shown to be significantly higher in MASLD patients, while the protective variant (rs72613567 T>TA) is associated with a decrease in fibrogenic pathways [10,13,14]. *MTARC1* is a molybdenum-containing enzyme that is localized to the outer mitochondrial membrane and has been reported to function in both drug detoxification and lipid metabolism [8,9]. The protective variant (rs264238 G>A) has been associated with decreased inflammation and fibrosis in MASLD [11,12]. Our understanding of how these genetic variants function in MASLD pathogenesis is lacking. Moreover, the impact of genetic variants on the levels of drug-metabolizing CYP enzymes in human hepatocytes has been largely unexplored.

This report describes the analysis of metabolic functions in human hepatocytes, including eighteen hepatic phase I/II activity studies, three CYP induction studies, and five studies on the inducibility and mRNA expression of CYP enzymes. Data from 126 human hepatocyte isolations are presented and classified by the presence of either high-risk or protection-associated MASLD genetic variants. These data provide an initial overview of the impact of genetic variants on drug-metabolizing enzymes in normal human hepatocytes, which could be helpful for drug discovery studies in the future.

## 2. Results

### 2.1. Study Design

To assess the role genetic variants might play in drug-metabolizing cytochrome P450 (CYP) enzymes, polymorphic sites associated with high-risk liver disease progression and protection were determined in 126 human hepatocytes. The cryopreserved primary hepatocytes were obtained from In Vitro ADMET Laboratories Inc. (IVAL, Columbia, MD, USA). The isolations were procured from human liver donors that were rejected for orthotopic liver transplantation. Liver tissue was equally distributed between males and females (M:F ratio, 1:1). The mean age was 44 years (range 0–77 years), while the ethnicity distribution of the processed specimens was 71.4% Caucasian, 16.7% Hispanic, 7.9% African American, and 3.9% other ethnic groups (e.g., Asian, Middle Eastern, or not provided). For all the cell isolation specimens, lower than 70% viability preparations were excluded from the study. We focused on five genetic variants in donors and recipients where robust GWAS have linked these variants to a spectrum of liver diseases ranging from steatosis to nonalcoholic steatohepatitis, hepatic fibrosis, hepatocarcinoma, ESLD, and increased risk of mortality in the general population [15]. These include *PNPLA3* rs738409, *MBOAT7* rs641738, *GCKR* rs780094, and two previously reported protective genetic variants *HSD17B13* rs72613567 and *MTARC1* rs2642438 (Table S1) [10,16,17,18,19,20,21,22]. We found that these variants were similarly distributed among the cases analyzed. The frequency of the gene variants was as follows: *PNPLA3* rs738409:G (minor homozygous), 6.35%; *MBOAT7* rs641738:T (minor homozygous), 21.5%; *GCKR* rs780094:T, 11.9%. The protective *HSD17B13* rs72613567:TA gene variant was present in 3.1% of the cases, while *MTARC1* rs2642438:A was present in 7.1% of the cases (Table S1). Test for Hardy–Weinberg equilibrium (HWE) was performed. The p-value for the variants was as follows: *PNPLA3* rs738409, *p* = 0.952; *MBOAT7* rs641738, *p* = 0.549; *GCKR* rs780094, *p* = 0.886; *HSD17B13* rs72613567, *p* = 0.659; and *MTARC1* rs2642438, *p* = 0.775. As the *p*-values were >0.05, there is not enough evidence to reject the null hypothesis that each SNP is in HWE.

### 2.2. SNPs and Liver Enzymes

**Figure 1:**
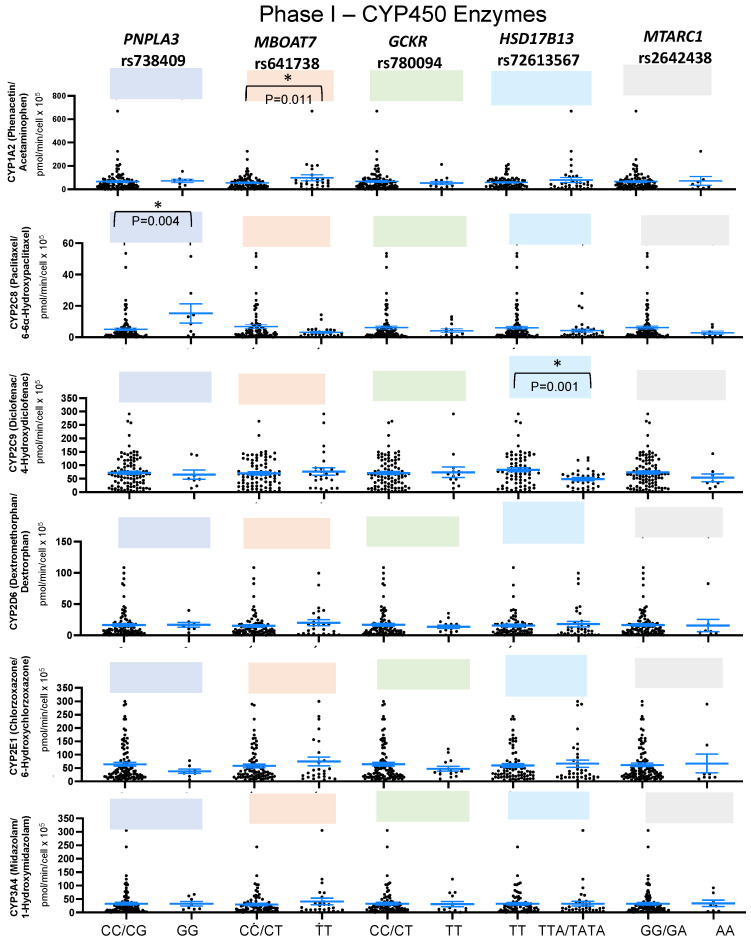
Functional association of variants in phase I CYP450 liver enzymes among healthy donors. The box plots represent minor homozygous and nonminor homozygous genotypes of *PNPLA3* rs738409 (blue), *MBOAT7* rs641738 (orange), *GCKR* rs780094 (green), *HSD17B13* rs72613567 (light blue), and *MTARC1* rs2642438 (gray) for CYP450 enzymes: CYP1A2 (n = 110), CYP2C8 (n = 105), CYP2C9 (n = 110), CYP2D6 (n = 108), CYP2E1 (n = 108), and CYP3A4 (n = 108). Each black dot represents a donor, and an unpaired two-sided Mann–Whitney U test with 95% CI was used to assess the difference in metabolite levels between minor homozygous and nonminor homozygous groups. The *p*-values are shown at the top of each box plot. * *p* < 0.05.

To investigate the extent to which the presence of these genetic variants might influence drug-metabolizing cytochrome P450 (CYP) enzyme activity, hepatic phase I activities were measured for several important cytochrome P450 family members, including CYPs 1A2, 2C8, 2C9, 2D6, 2E1, and 3A4 (Table 1). Interestingly, we found significant differences in CYP1A2 activity (*p* = 0.011) in human hepatocytes with *MBOAT7* minor homozygous (TT) compared to nonminor homozygous (CC/CT) using the substrate phenacetin and the metabolite acetaminophen and in CYP2C8 activity (*p* = 0.004) in human hepatocytes with *PNPLA3* minor homozygous (GG) compared to nonminor homozygous (CC/CG) using the substrate paclitaxel and the metabolite 6-6α-hydroxypaclitaxel (Figure 1 and Table S2).

Moreover, it was also observed that the activity of CYP2C9 (*p* = 0.001) using the substrate diclofenac and the metabolite 4-hydroxydiclofenac was significantly lower in human hepatocytes with *HSD17B13* minor homozygous (TTA/TATA) compared to nonminor homozygous (TT) (Figure 1 and Table S2).

Given that CYP450 enzymes exhibit substantial polymorphism, we verified whether these significant differences observed in the results of CYP1A2, CYP2C8, and CYP2C9 could be attributed to the specific genetic variants under investigation rather than generic CYP450 polymorphisms. For this purpose, we conducted a genotyping test for one polymorphism for each CYP450 enzyme: *CYP1A2* rs762551, *CYP2C8* rs11572080, and *CYP2C9* rs1057910. When comparing individuals carrying minor homozygous for the *MBOAT7* rs641738, *PNPLA3* rs738409, and *HSD17B13* rs72613567 variants and minor homozygous of CYP450 polymorphism, we found no significant difference (*p* = 0.6052, *p* = not enough sample to evaluate, and *p* = 0.1929, respectively). We also compared individuals carrying nonminor homozygous for *MBOAT7* rs641738, *PNPLA3* rs738409, and *HSD17B13* rs72613567 variants and carrying nonminor homozygous of CYP450 polymorphism, and we found no significant difference (*p* = 0.452, *p* = 0.4304, and *p* = 0.1331, respectively). These results confirm that the heightened activity observed was indeed attributed to the specific variant under examination and not because of CYP polymorphism (Figure S1).

On the other hand, there was no significant difference in activity of CYP2D6, CYP2E1, and CYP3A4 between human hepatocytes carrying genetic variants for *PNPLA3* rs738409, *MBOAT7* rs641738, *GCKR* rs780094, *HSD17B13* rs72613567, and *MTARC1* rs2642438 (Figure 1 and Table S2).

**Figure 2:**
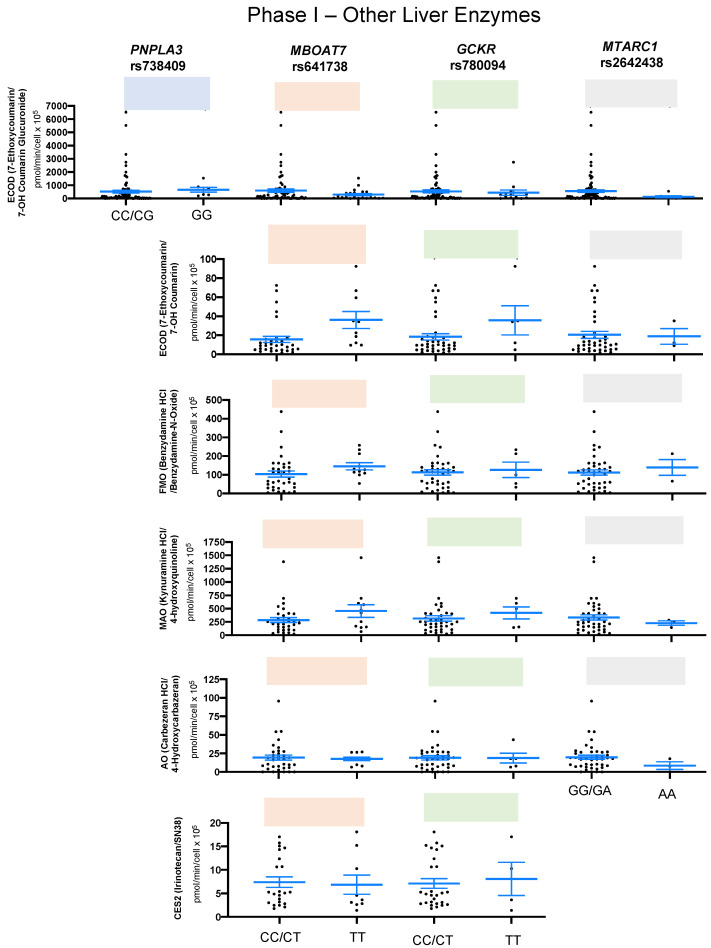
Association of variants in phase I liver enzymes among healthy donors. The box plots represent the functional assay showing minor homozygous and nonminor homozygous genotypes of *PNPLA3* rs738409 (blue), *MBOAT7* rs641738 (orange), *GCKR* rs780094 (green), *HSD17B13* rs72613567 (light blue), and *MTARC1* rs2642438 (gray) for phase I liver enzymes: ECOD (n = 99), ECOD (n = 43), FMO (n = 45), MAO (n = 44), AO (n = 45), and CES2 (n = 31). Each black dot represents a donor, and an unpaired two-sided Mann–Whitney U test with 95% CI was used to assess the difference in metabolite levels between minor homozygous and nonminor homozygous groups. The *p*-values are shown at the top of each box plot.

Additionally, others phase I enzymes were analyzed in human hepatocytes carrying genetic variants for *PNPLA3* rs738409, *MBOAT7* rs641738, *GCKR* rs780094, and *MTARC1* rs2642438, and no significant difference was found in the activity of ECOD (substrate: 7-ethoxycoumarin; marker metabolite: 7-OH coumarin glucuronide), ECOD (substrate: 7-ethoxycoumarin; marker metabolite: 7-OH coumarin), FMO (substrate: benzydamine HCl; marker metabolite: benzydamine-N-oxide), MAO (substrate: kynuramine HCl; marker metabolite: 4-hydroxyquinoline), AO (substrate: carbezeran HCl; marker metabolite: 4-hydroxycarbazeran), or CES2 (substrate: irinotecan; marker metabolite: SN38) (Figure 2 and Table S3).

Among the metabolic pathways that can directly affect the integrity of not only the liver but also multiple other organs are phase II conjugation enzymes. The phase II detoxification enzymes comprise conjugation reactions that facilitate the elimination of harmful metabolites from the body, thus reducing their toxicity. The enzymes involved in these processes include sulfotransferases (SULT), UDP-glucuronosyltransferases (UGT), glutathione S-transferases (GST), and N-acetyltransferases (NAT) [23].

**Figure 3:**
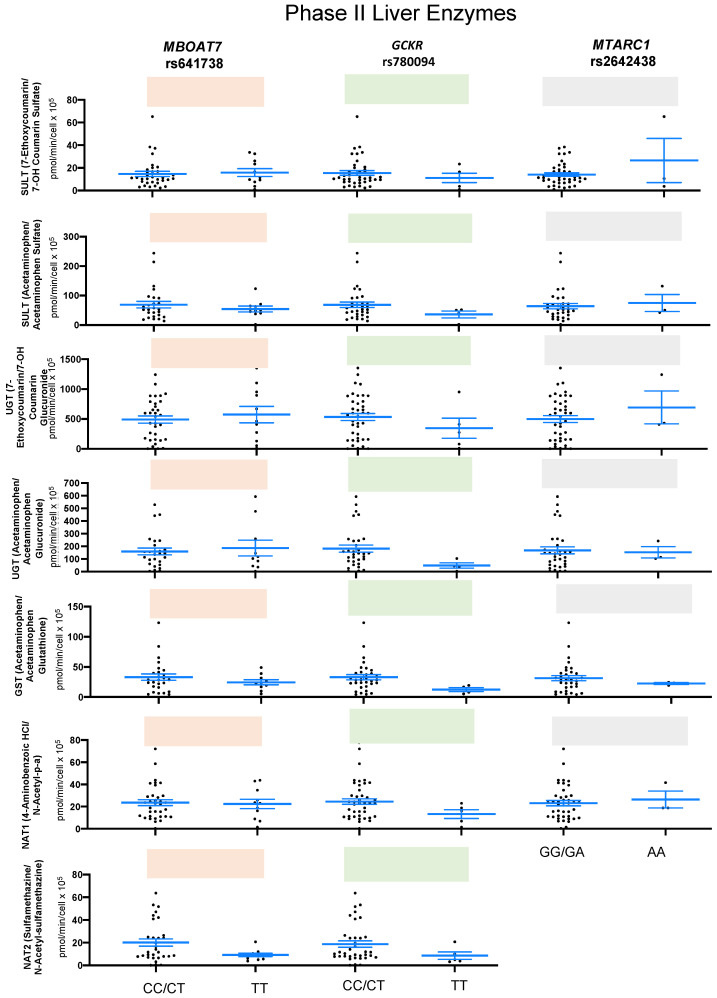
Functional assay showing the association of variants in phase II enzymes among healthy donors. The box plots represent minor homozygous and nonminor homozygous genotypes of *MBOAT7* rs641738 (orange), *GCKR* rs780094 (green), and *MTARC1* rs2642438 (gray) for phase I liver enzymes: SULT (n = 44), SULT (n = 36), UGT (n = 44), GST (n = 36), NAT1 (n = 45), and NAT2 (n = 41). Each black dot represents a donor, and an unpaired two-sided Mann–Whitney U test with 95% CI was used to assess the difference in metabolite levels between minor homozygous and nonminor homozygous groups. The *p*-values are shown at the top of each box plot.

When phase II liver enzyme activity were measure in human hepatocytes carrying *MBOAT7* rs641738, *GCKR* rs780094, *MTARC1* rs2642438, and controls, none of the genetic variants demonstrated a significant activity for SULT (substrate: 7-ethoxycoumarin; marker metabolite: 7-OH coumarin sulfate), SULT (substrate: acetaminophen; marker metabolite: acetaminophen sulfate), UGT (substrate: 7-ethoxycoumarin; marker metabolite: 7-OH coumarin glucuronide), UGT (substrate: acetaminophen; marker metabolite: cetaminophen glucuronide), GST (substrate: acetaminophen; marker metabolite: acetaminophen glutathione), NAT1 (substrate: 4-aminobenzoic HCl; marker metabolite: N-acetyl-p-a), and NAT2 (substrate: sulfamethazine; marker metabolite: N-acetyl-sulfamethazine) (Figure 3 and Table S4). Additionally, CYP450 induction-mediated interactions are always a major interest in clinical practice as many patients undergo multidrug therapies. CYP450 induction of a metabolizing enzyme is due to de novo CYP450 protein synthesis after drug exposure [23]. Thus, to investigate whether genetic variants affect CYP450 inducibility, human hepatocytes carrying *PNPLA3* rs738409, *MBOAT7* rs641738, or *GCKR* rs780094 were treated with omeprazole to induce CYP1A2, phenobarbital to induce CYP2B6, and rifampicin to induce CYP3A4.

**Figure 4:**
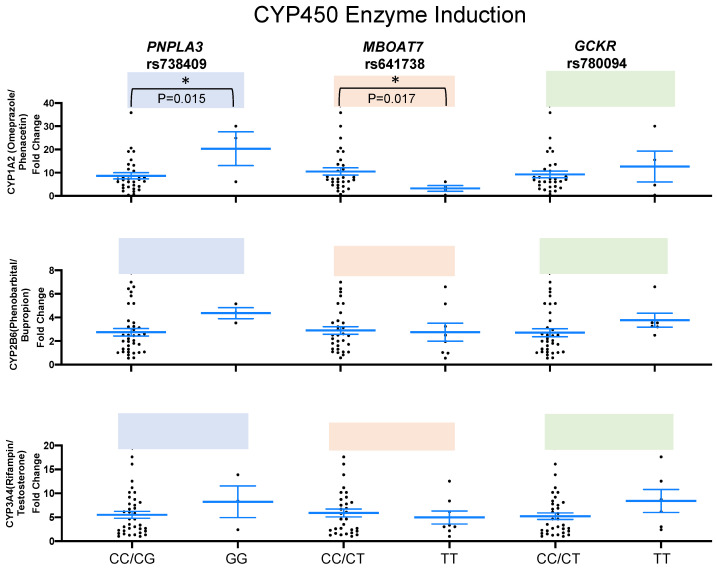
Phase I CYP450 enzymes induction among healthy donors. The box plots represent minor homozygous and nonminor homozygous genotypes of *PNPLA3* rs738409 (blue), *MBOAT7* rs641738 (orange), and *GCKR* rs780094 (green) for phase I CYP450 liver enzymes: CYP1A2 (n = 34), CYP2B6 (n = 39) and CYP3A4 (n = 39). Each black dot represents a donor, and an unpaired two-sided Mann–Whitney U test with 95% CI was used to assess the difference in enzyme induction levels between minor homozygous and nonminor homozygous groups. The *p*-values are shown at the top of each box plot. * *p* < 0.05.

**Figure 5:**
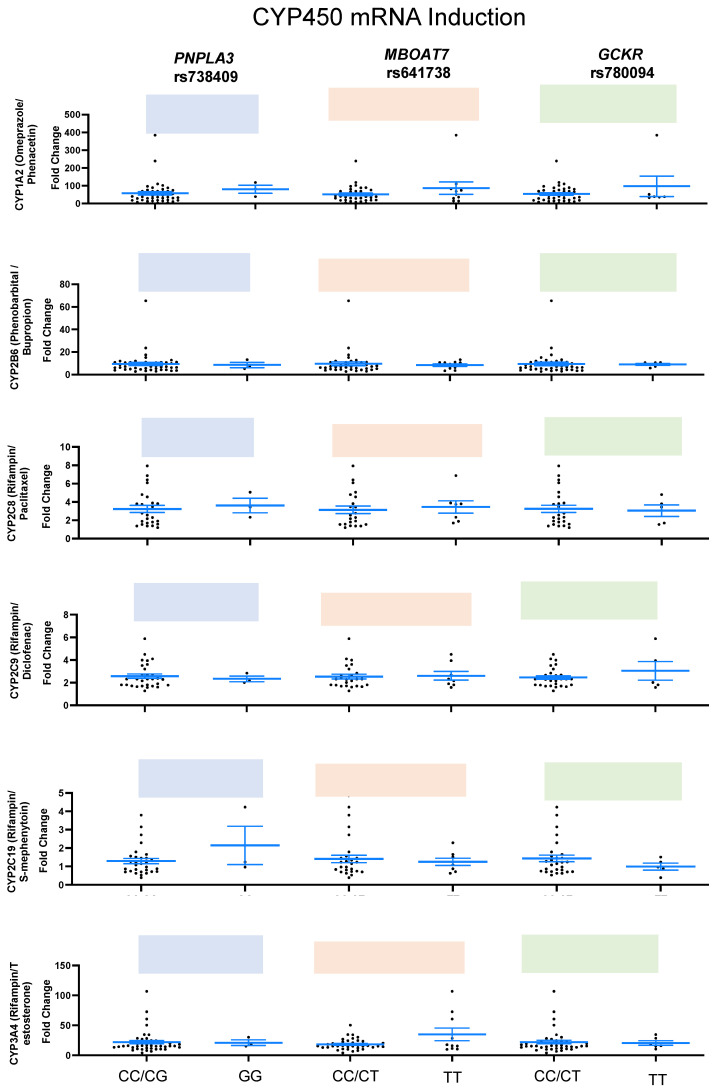
mRNA induction of phase I CYP450 enzymes among healthy donors. The box plots represent minor homozygous and nonminor homozygous genotypes of *PNPLA3* rs738409 (blue), *MBOAT7* rs641738 (orange), and *GCKR* rs780094 (green) for mRNA induction levels of CYP450 liver enzymes: CYP1A2 (n = 47), CYP2B6 (n = 47), CYP2C8 (n = 29). CYP2C9 (n = 33), CYP2C19 (n = 33) and CYP3A4 (n = 47). Each black dot represents a donor, and an unpaired two-sided Mann–Whitney U test with 95% CI was used to assess the difference in mRNA induction levels between minor homozygous and nonminor homozygous groups. The *p*-values are shown at the top of each box plot.

We found significant difference in CYP1A2 activity in human hepatocytes with *PNPLA3* (*p* = 0.015) minor homozygous (GG) compared to nonminor homozygous (CC/CG) and with *MBOAT7* (*p* = 0.017) minor homozygous (TT) compared to nonminor homozygous (CC/CT). There were no significant differences in CYP2B6 and CYP3A4 induction in any of the variants (Figure 4 and Table S5). Similarly, mRNA levels of CYP1A, 2B6, 2C8, 2C9, C19, and 3A4 were measured, and no significant differences were found in the mRNA levels after drug exposure (Figure 5 and Table S6).

## 3. Discussion

In this study, we genetically profiled 126 healthy liver donors for five different genetic variants previously associated with predisposition to the development of liver disease (*PNPLA3* rs738409, *GCKR* rs780094, and *MBOAT7* rs641738) or protection of liver disease (*HSD17B13* rs72613567 and *MTARC1* rs2642438) [24,25,26,27,28]. We analyzed minor and nonminor homozygous in the genotype frequencies to determine associations between these genetic variants and phase I and II liver enzyme activity and inducibility.

It is already known that CYP450 are highly polymorphic. For that reason and to guarantee that the significant results observed was due to the variants and not because of CYP450 polymorphism, we performed genotyping tests for the most cited polymorphism related to CYP1A2 using the substrate phenacetin, CYP2C8 using the substrate paclitaxel, and CYP2C9 using diclofenac as the substrate. No significant difference was found in any of the hepatocytes carrying the CYPs mutant allele (minor homozygous) and nonminor homozygous that also carries minor homozygous and nonminor homozygous *MBOAT7* rs641738, *PNPLA3* rs738409, and *HSD17B13* rs72613567 variants [29,30,31].

We already know that elevated hepatic enzyme activity can potentially reduce pharmacotherapeutic effectiveness, leading to toxic liver disease. Consequently, the reduced metabolization capacity of phase II enzymes may lead to toxic consequences when using clinically prescribed medications.

As demonstrated previously for *PNPLA3* rs738409, the presence of allele GG is linked to escalated liver enzyme levels and an increased susceptibility to liver toxicity compared to the CC allele. This risk is particularly notable when individuals are treated with medications such as cyclophosphamide, cytarabine, daunorubicin, doxorubicin, mercaptopurine, methotrexate, aspargase, thioguanine, and vincristine. Our research indicated that the *PNPLA3* rs738409 variant increased CYP2C8 activity when assessed with paclitaxel and 7-ethoxycoumarin as substrates [32]. Additionally, we observed that the *MBOAT7* rs641738 variant was associated with an increase in CYP1A2 activity, while the presence of *HSD17B13* rs72613567 led to a reduction in CYP2C9 activity [32].

This potentially translates to an increased risk of adverse effects due to reduced metabolization capacity of phase II enzymes when individuals are prescribed clinical medications. According to the literature, the *GCKR* allele T is linked to an increased response to fenofibrate medication in individuals with hypertriglyceridemia. Interestingly, our data did not indicate a significant association between the *GCKR* rs780094 genetic variant and any significant increase or decrease in liver enzyme levels [33].

Our studies highlight the potential association between metabolic and toxicological consequences that certain genetic variants may present in human hepatocytes in human livers. Most of these variants cause harmful effects of elevated levels of lipids (lipotoxicity) on cells and tissues, particularly when these lipids accumulate to excessive levels.

Lipotoxicity in the liver can cause inflammation, oxidative stress, and cell damage, leading to MASLD, which ranges from simple fat accumulation (steatosis) to more severe conditions such as nonalcoholic steatohepatitis (MASH) and cirrhosis [34].

It is essential to study the relationship between P450 levels and MASLD to better understand the underlying mechanisms that contribute to the disease. This study can potentially lead to the development of targeted therapies or interventions aimed at modulating P450 enzymes to manage or prevent MASLD. However, it is important to note that the exact mechanisms and specific roles of P450 enzymes in MASLD may vary depending on individual genetic factors, environmental influences, and overall metabolic health.

Most of the gene variant associations examined here have been studied, and lipid droplet biology, intracellular lipid synthesis and degradation, and secretion of very-low-density lipoproteins [15] have been found to play a role in the potential mechanisms behind the development of MASLD, although the mechanisms by which the variants lead to ESLD and cellular death are poorly understood.

About 90% of medications are processed by multiple human phase I enzymes that regularly participate in clinically relevant drug–drug interactions. By activating or inhibiting phase I enzymes, certain drugs can either speed up or slow down the metabolism of other drugs [35]. On the other hand, enzymes from phase II are crucial for the metabolic inactivation of pharmacologically active substances as well as the biotransformation of endogenous substances and xenobiotics into more excretable forms [36].

Drug-induced liver injury (DILI) is a significant concern as it can result in mild and reversible liver dysfunction turning to severe and life-threatening conditions, such as acute liver failure. When investigating the role of DILI in MASLD, studies that employ RUCAM causality assessment provide valuable data to establish the association between specific drugs and liver injury, aiding in the safe prescribing and management of patients with metabolic-associated liver diseases [37,38,39].

In 2018, Rolf Teschke collected data from DILI cases based on the RUCAM system from international databases. A total of 3312 drugs were analyzed, with amoxicillin–clavulanate (an antibiotic), flucloxacillin (an antibiotic), atorvastatin (a lipid-lowering agent), disulfiram (a substance abuse agent), and diclofenac (an NSAID) being the five most relevant medications related to DILI [40].

Patients with MASLD may already have underlying liver abnormalities due to fatty liver, inflammation, or fibrosis. They also often share risk factors, such as obesity, diabetes, and metabolic syndrome [41]. When exposed to certain drugs, these patients may be more susceptible to DILI as their liver might have reduced resilience to handle additional stressors when certain drugs are introduced, thereby exacerbating liver injury [42].

We observed that patients who were minor homozygous for genetic variants previously related to predisposition to liver disease (*PNPLA3* and *MBOAT7*) had increased phase I enzyme activity. As opposed to that, the *HSD17B13* variant, which is related to protective effect in liver disease, led to a decrease in phase I enzyme activity. The relationship between phase I enzymes and pharmacotherapy is already known. Increased hepatic phase I activity has the potential to decrease the pharmacotherapeutic efficacy of recognized substrates by increasing their metabolism or increasing the production of reactive metabolites and oxidative stress [23,43]. Thus, the lower capacity of metabolization of phase II enzymes can bring toxic consequences from clinically utilized medications [36]. Conversely, decreased phase I enzyme activity also decreases the production of reactive metabolites and oxidative stress, as we observed in *HSD17B13*.

The current study on drug-metabolizing cytochrome P450 (CYP) enzymes identified the importance of additional potential mechanisms of cellular stress that, together with environmental factors and unexplored mechanisms of disease, may facilitate disease progression. This study is limited by the small number of human hepatocyte cases that were analyzed carrying relatively uncommon genetic variants. Thus, future studies will be dedicated to expanding the number of cases to improve the power of the current analysis. In summary, the studies described here highlight pharmacogenomic interactions and help to expand understanding of human liver diseases and preventive therapies, paving the way for precision medicine.

## 4. Material and Methods

This study analyzed metabolic activity and enzyme induction of liver enzymes in healthy liver donors (n = 126). Supplementary Table S1 presents the detailed characteristics of the cohorts. Fresh isolated primary hepatocytes were obtained from healthy liver donors, and liquid chromatography–mass spectrometry (LC–MS) was performed. Metabolic activities were obtained for selected phase I and phase II liver enzymes by LC–MS. Table 1 presents the metabolic pathways, substrates with their concentration, the marker metabolites analyzed, iron mode applications, and mass transition monitoring. Genotyping was also performed using genomic DNA extracted from hepatocyte samples. For the cohorts, participants were classified into minor homozygotes and nonminor homozygotes (major homozygotes + heterozygotes) for the five investigated gene polymorphisms (*PNPLA3* rs738409, *MBOAT7* rs641738, *GCKR* rs780094, *HSD17B13* rs72613567, and *MTARC1* rs2642438) (Table S1).

### 4.1. Human Primary Hepatocytes

Cryopreserved primary hepatocytes (n = 126) from healthy donors were obtained from In Vitro ADMET Laboratories Inc. (IVAL, Columbia, MD, USA). These hepatocytes were isolated from liver specimens of donors who were negative for hepatitis C virus (HCV), hepatitis B virus (HBV), and human immunodeficiency virus (HIV).

### 4.2. DNA Isolation and Genotyping

Genomic DNA was isolated from the isolated hepatocytes using the DNeasy Blood and Tissue Kit (QIAGEN, Hilden, Germany) following the manufacturer’s instructions. DNA quantity and quality were measured using a NanoDrop Lite spectrometer (ThermoFisher Scientific, Waltham, MA, USA). Genotyping reactions containing 1× TaqMan™ Genotyping Master Mix (Applied Biosystems, Foster City, CA, USA), 1× TaqMan^®^ Genotyping Assays (Applied Biosystems, Foster City, CA, USA), and 4.5 pg of genomic DNA were prepared in MicroAmp Fast Optical 96-well plates (Applied Biosystems, Foster City, CA, USA). Real-time PCR was performed using the StepOnePlus system (Applied Biosystems, Foster City, CA, USA).

In order to confirm that the significant differences observed in the results of the enzyme inductions and the variability in the mutations of CYP1A2, CYP2C8, and CYP2C9 were due to the tested genetic variants and not due to a CYP450 polymorphism when comparing individuals with minor and non-minor homozygous for *MBOAT7* rs641738, *PNPLA3* rs738409, and *HSD17B13* rs72613567, we performed the genotyping for CYP1A2 rs762551, CYP2C9 rs1057910and CYP2C8 rs11572080. Details of the TaqMan^®^ Genotyping Assays are listed in Table S7.

### 4.3. Drug Metabolism Studies

Chemicals: Dextrorphan tartrate, diclofenac sodium salt, 4-hydroxydiclofenac, S-mephenytoin, 4-hydroxyquinoline, paclitaxel, and testosterone were purchased from Cayman Chemical (Ann Arbor, MI, USA). 7-Hydroxycoumarin was purchased from Chem Service (West Chester, PA, USA). Benzydamine N-oxide, 7-hydroxycoumarin sulfate potassium salt, kynuramine hydrobromide, and N-acetyl sulfamethazine were obtained from Santa Cruz Biotechnology (Dallas, TX, USA). 4-Acetamidobenzoic acid, p-acetamidophenyl β-D-glucuronide sodium salt, 4-aminobenzoic acid, benzydamine hydrochloride, chlorzoxazone, coumarin, dextromethorphan hydrobromide, 6β-hydroxytestosterone, 7-hydroxycoumarin β-D-glucuronide sodium salt, 7-ethoxycoumarin, paracetamol sulfate potassium, phenacetin, and sulfamethazine were purchased from Sigma Aldrich (St. Louis, MO, USA). Carbazeran, 4-hydroxycarbazeran, 6-hydroxychlorzoxazone, 6α-hydroxypaclitaxel, acetaminophen glutathione disodium salt, midazolam, 1′-hydroxymidazolam, and 4-hydroxy-S-mephenytoin were obtained from Toronto Research Chemicals (Toronto, Canada). All other drug-metabolizing enzyme substrates were obtained from Sigma Aldrich (St. Louis, MO, USA).

Incubation with DME substrates: All incubations were performed in 96-well cell culture plates (Falcon, obtained from VWR Inc., West Chester, PA, USA). The cryopreserved human hepatocytes were thawed in a 37 °C water bath, with 1 mL of the thawed suspension added to 50 mL of universal cryopreservation recovery medium (UCRM, In Vitro ADMET Laboratories Inc. (IVAL), Columbia, MD, USA) in a 50 mL conical cell culture tube (Falcon, obtained from VWR Inc.) and centrifuged at 100× *g* for 10 min. The cell pellet from each conical tube was resuspended in 4 mL of hepatocyte incubation medium (HQM, IVAL) for viability determination (Trypan blue exclusion) and cell concentration determination. The cell suspension was then adjusted with HQM to 2× of the final cell density. All DME substrates were prepared in HQM at 2× of the final concentrations and added at a volume of 50 µL per well in a 96-well cell culture plate. The hepatocyte and substrate plates were prewarmed to 37 °C for 15 min in a cell culture incubator before the initiation of incubation by pipetting 50 µL of hepatocytes into each well of the 96-well plates containing the substrates and returned to the cell culture incubator without shaking for the incubation duration of 30 min. At the end of each incubation, 100 µL of acetonitrile was added into each well to terminate metabolism. The plates after termination were stored in a −80 °C freezer for later LC/MS–MS quantification of metabolite formation.

LC/MS/MS Analysis: Upon thawing, an aliquot of 200 µL of each sample was transferred from each well into a labeled 96-well plate, followed by the addition of 100 µL of acetonitrile solution containing the internal standard tolbutamide (250 nM) and mixing. All samples were centrifuged at 3500 rpm for 5 min. An aliquot of 100 µL of supernatant from each sample was transferred to a 96-well plate and diluted with 200 µL of deionized water by mixing before LC/MS/MS analysis. CYP1A2, CYP2A6, CYP2B6, CYP2C8, CYP2C9, CYP2C19, CYP2D6, CYP2E1, CYP3A4 (midazolam 1′-hydroxylation), CYP3A4 (testosterone 6b-hydroxylation), ECOD, UGT, SULT, GST, FMO, MAO, AO, NAT1, and NAT2 metabolites were quantified using API 4000 QTRAP mass spectrometer with an electrospray ionization source (AB SCIEX, Framingham, MA, USA) connected to an Agilent 1200 series HPLC (Agilent Technologies, Santa Clara, CA, USA) using LC/MS/MS MRM mode, and the mass transitions (parent to daughter ion) were monitored (Table 1). An Agilent Zorbax Eclipse Plus C18 column (4.6 × 75 mm i.d., 3.5 µm; Agilent Technologies, Santa Clara, CA, USA) at a flow rate of 1 mL/min was used for the chromatography separation. The mobile phase consisted of 0.1% formic acid in acetonitrile (A) and 0.1% formic acid in water (B). The gradient for the positive ion mode operation was programed as follows: 0 to 2.5 min, increase B from 5 to 95%; 2.5 to 3.5 min, 95% B; 3.5 to 3.6 min, decrease B to 5%; run time, 5 min. The gradient program for the negative ion mode was follows: 0 to 3 min, increase B from 5 to 95%; 3 to 4 min, 95% B; 4 to 4.2 min, decrease B to 5%; run time, 6 min. Data acquisition and data processing were performed with the software Analyst 1.6.2 (AB SCIEX, Framingham, MA, USA).

CYP450 inducibility: The hepatocytes were treated with omeprazole (100 μM), phenobarbital (1000 μM), and rifampicin (20 μM) 24 h after plating for a duration of 48 h, with the treatment medium refreshed after the first 24 h of treatment.

Data Analysis: Data are presented as mean and standard deviation of triplicate incubations derived using the Microsoft Excel 6.0 software. Statistical analysis was performed using the Student’s *t*-test with the Microsoft Excel 6.0 software, with the probability of *p* < 0.05 considered as statistically significant. Specific activity (pmol/min/million hepatocytes) of each drug-metabolizing enzyme pathway was determined by dividing the total metabolite formed by the incubation time and normalized to cell concentration.

### 4.4. Statistical Methods

The data were analyzed when at least 3 or more data cases in each group were available. Correlation, multivariate, and postestimation analyses were performed using Stata/SE v18.0. After univariate analysis, pairwise correlation between independent variables was assessed using the *pwcorr* command with Bonferroni adjustment at a significance level of 0.05. All pairs of independent variables were not significantly correlated. Linear regression was then performed using the *regress* command with the activity/inducibility/mRNA level of each drug-metabolizing enzyme as the dependent variable and the genotypes for the five SNPs, age, and sex as the independent variables. After linear regression, heteroskedasticity was assessed using the *ivhettest*, *nr2* command, a Lagrange multiplier version of the Breusch–Pagan test identified by Wooldredge that returns the standard White/Koenker nR-sq test statistic for heteroskedasticity. Heteroskedasticity was not observed in the dataset. Multicollinearity was also assessed by determining variance inflation factor (VIF) for each of the independent variables using the *estat vif* function. All VIFs were less than 2.5, suggesting the absence of multicollinearity. To account for multiple testing, significant *p*-values were determined following the Benjamini and Hochberg false discovery rate (FDR) method. The *p*-value for each enzyme is described in Tables S2–S6, and the ones where we observed significant differences are highlighted in blue and noted as significant in the notes of the table [44,45,46].

Test for Hardy–Weinberg equilibrium (HWE) was performed using Stata/SE v18.0. HWE for each SNP was assessed using the *hwsnp* command at a significance level of 0.05.

## Figures and Tables

**Table 1:** LCMS conditions for the quantification of metabolites from drug-metabolizing enzyme-selective pathways.

Metabolic Pathway	Substrate and Concentrations	Marker Metabolite Analyzed	Ion Mode Application	Mass Transitions Monitoring
CYP1A2	Phenacetin (100 μM)	Acetaminophen	Positive	*m*/*z* 152.1 to 109.9
CYP2A6	Coumarin (50 μM)	7-Hydroxycoumarin; 7-hydroxycoumarin glucuronide; 7-hydroxycoumarin sulfate	Negative	*m*/*z* 161.0 to 132.9 (7-hydroxycoumarin); *m*/*z* 336.9 to 160.9 (glucuronide); *m*/*z* 240.9 to 161.0 (sulfate)
CYP2B6	Bupropion (500 μM)	Hydroxybupropion	Positive	*m*/*z* 250.1 to 130.1
CYP2C8	Paclitaxel (20 μM)	6α-Hydroxypaclitaxel	Positive	*m*/*z* 870.4 to 525.2
CYP2C9	Diclofenac (25 μM)	4-Hydroxydiclofenac	Negative	*m*/*z* 309.8 to 265.9
CYP2C19	S-Mephenytoin (250 μM)	4-Hydroxy-S-mephenytoin	Positive	*m*/*z* 235.2 to 150.0
CYP2D6	Dextromethorphan (15 μM)	Dextrorphan	Positive	*m*/*z* 258.1 to 157.1
CYP1A2	Acetaminophen (100 μM)	Acetaminophen	Positive	*m*/*z* 152.1 to 109.9
CYP2B6	Bupropion (500 μM)	Hydroxybupropion	Positive	*m*/*z* 250.1 to 130.1
CYP3A4	Testosterone (200 μM)	6β-Hydroxytestosterone	Positive	*m*/*z* 305.2 to 269.1
CYP2E1	Chlorzoxazone (250 μM)	6-Hydroxychlorzoxazone	Negative	*m*/*z* 183.9 to 119.8
CYP3A4	Midazolam (20 μM)	1′-Hydroxymidazolam	Positive	*m*/*z* 342.1 to 203.1
CYP3A4	Testosterone (200 μM)	6β-Hydroxytestosterone	Positive	*m*/*z* 305.2 to 269.1
ECOD	7-Ethoxycoumarin (100 μM)	7-Hydroxycoumarin	Negative	*m*/*z* 161.0 to 132.9
UGT	7-Hydroxycoumarin (100 μM)	7-Hydroxycoumarin glucuronide	Negative	*m*/*z* 336.9 to 160.9
SULT	7-Hydroxycoumarin (100 μM)	7-Hydroxycoumarin sulfate	Negative	*m*/*z* 240.9 to 161.0
UGT	Acetaminophen (10, 100, 200 mM)	Acetaminophen glucuronide	Negative	*m*/*z* 326.0 to 150.0
SULT	Acetaminophen (10, 100, 200 mM)	Acetaminophen sulfate	Negative	*m*/*z* 229.8 to 150.0
GST	Acetaminophen (10, 100, 200 mM)	Acetaminophen glutathione	Negative	*m*/*z* 455.0 to 271.8
FMO	Benzydamine hydrochloride (250 μM)	Benzydamine-N-oxide	Positive	*m*/*z* 326.4 to 102.1
MAO	Kynuramine hydrobromide (160 μM)	4-Hydroxyquinoline	Negative	*m*/*z* 144.1 to 102.1
AO	Carbazeran (10 μM)	4-Hydroxycarbazeran	Positive	*m*/*z* 377.0 to 234.2
NAT1	4-Aminobenzoic acid (200 μM)	N-Acetyl-p-aminobenzoic acid	Negative	*m*/*z* 178.0 to 133.7

## Data Availability

Data is contained within the article or Supplementary Material.

## Supplementary Materials

The supporting information can be downloaded at: https://www.mdpi.com/article/10.3390/ijms241713406/s1.

## References

[1] Estes C., Razavi H., Loomba R., Younossi Z., Sanyal A.J. (2018). Modeling the epidemic of nonalcoholic fatty liver disease demonstrates an exponential increase in burden of disease. Hepatology.

[2] Satapathy S.K., Sanyal A.J. (2015). Epidemiology and Natural History of Nonalcoholic Fatty Liver Disease. Semin. Liver Dis..

[3] Pacana T., Sanyal A.J. (2015). Recent advances in understanding/management of non-alcoholic steatohepatitis. F1000Prime Rep..

[4] Jain M.R., Giri S.R., Trivedi C., Bhoi B., Rath A., Vanage G., Vyas P., Ranvir R., Patel P.R. (2015). Saroglitazar, a novel PPARalpha/gamma agonist with predominant PPARalpha activity, shows lipid-lowering and insulin-sensitizing effects in preclinical models. Pharmacol. Res. Perspect..

[5] Speliotes E.K., Yerges-Armstrong L.M., Wu J., Hernaez R., Kim L.J., Palmer C.D., Gudnason V., Eiriksdottir G., Garcia M.E., Launer L.J. (2011). Genome-wide association analysis identifies variants associated with nonalcoholic fatty liver disease that have distinct effects on metabolic traits. PLoS Genet..

[6] Schulze K., Imbeaud S., Letouzé E., Alexandrov L.B., Calderaro J., Rebouissou S., Couchy G., Meiller C., Shinde J., Soysouvanh F. (2015). Exome sequencing of hepatocellular carcinomas identifies new mutational signatures and potential therapeutic targets. Nat. Genet..

[7] Pingitore P., Pirazzi C., Mancina R.M., Motta B.M., Indiveri C., Pujia A., Montalcini T., Hedfalk K., Romeo S. (2014). Recombinant PNPLA3 protein shows triglyceride hydrolase activity and its I148M mutation results in loss of function. Biochim. Biophys. Acta.

[8] Thangapandi V.R., Knittelfelder O., Brosch M., Patsenker E., Vvedenskaya O., Buch S., Hinz S., Hendricks A., Nati M., Herrmann A. (2021). Loss of hepatic Mboat7 leads to liver fibrosis. Gut.

[9] Fernandes Silva L., Vangipurapu J., Kuulasmaa T., Laakso M. (2019). An intronic variant in the GCKR gene is associated with multiple lipids. Sci. Rep..

[10] Abul-Husn N.S., Cheng X., Li A.H., Xin Y., Schurmann C., Stevis P., Liu Y., Kozlitina J., Stender S., Wood G.C. (2018). A Protein-Truncating *HSD17B13* Variant and Protection from Chronic Liver Disease. N. Engl. J. Med..

[11] Emdin C.A., Haas M.E., Khera A.V., Aragam K., Chaffin M., Klarin D., Hindy G., Jiang L., Wei W.Q., Feng Q. (2020). A missense variant in Mitochondrial Amidoxime Reducing Component 1 gene and protection against liver disease. PLoS Genet..

[12] Hudert C.A., Adams L.A., Alisi A., Anstee Q.M., Crudele A., Draijer L.G., Furse S., Hengstler J.G., Jenkins B., EU-PNAFLD Investigators (2022). Variants in mitochondrial amidoxime reducing component 1 and hydroxysteroid 17-beta dehydrogenase 13 reduce severity of nonalcoholic fatty liver disease in children and suppress fibrotic pathways through distinct mechanisms. Hepatol. Commun..

[13] Ma Y., Belyaeva O.V., Brown P.M., Fujita K., Valles K., Karki S., de Boer Y.S., Koh C., Chen Y., Du X. (2019). 17-Beta Hydroxysteroid Dehydrogenase 13 Is a Hepatic Retinol Dehydrogenase Associated with Histological Features of Nonalcoholic Fatty Liver Disease. Hepatology.

[14] Motomura T., Amirneni S., Diaz-Aragon R., Faccioli L.A., Malizio M.R., Coard M.C., Kocas-Kilicarslan Z.N., Frau C., Haep N., Ostrowska A. (2021). Is *HSD17B13* Genetic Variant a Protector for Liver Dysfunction? Future Perspective as a Potential Therapeutic Target. J. Pers. Med..

[15] Trepo E., Valenti L. (2020). Update on NAFLD genetics: From new variants to the clinic. J. Hepatol..

[16] Rotman Y., Koh C., Zmuda J.M., Kleiner D.E., Liang T.J., Nash C.R.N. (2010). The association of genetic variability in patatin-like phospholipase domain-containing protein 3 (*PNPLA3*) with histological severity of nonalcoholic fatty liver disease. Hepatology.

[17] Buch S., Stickel F., Trepo E., Way M., Herrmann A., Nischalke H.D., Brosch M., Rosendahl J., Berg T., Ridinger M. (2015). A genome-wide association study confirms *PNPLA3* and identifies *TM6SF2* and MBOAT7 as risk loci for alcohol-related cirrhosis. Nat. Genet..

[18] Barata L., Feitosa M.F., Bielak L.F., Halligan B., Baldridge A.S., Guo X., Yerges-Armstrong L.M., Smith A.V., Yao J., Palmer N.D. (2019). Insulin Resistance Exacerbates Genetic Predisposition to Nonalcoholic Fatty Liver Disease in Individuals Without Diabetes. Hepatol. Commun..

[19] Kozlitina J., Smagris E., Stender S., Nordestgaard B.G., Zhou H.H., Tybjærg-Hansen A., Vogt T.F., Hobbs H.H., Cohen J.C. (2014). Exome-wide association study identifies a *TM6SF2* variant that confers susceptibility to nonalcoholic fatty liver disease. Nat. Genet..

[20] Liu Y.L., Reeves H.L., Burt A.D., Tiniakos D., McPherson S., Leathart J.B., Allison M.E., Alexander G.J., Piguet A.C., Anty R. (2014). *TM6SF2* rs58542926 influences hepatic fibrosis progression in patients with non-alcoholic fatty liver disease. Nat. Commun..

[21] Romeo S., Kozlitina J., Xing C., Pertsemlidis A., Cox D., Pennacchio L.A., Boerwinkle E., Cohen J.C., Hobbs H.H. (2008). Genetic variation in *PNPLA3* confers susceptibility to nonalcoholic fatty liver disease. Nat. Genet..

[22] Sveinbjornsson G., Ulfarsson M.O., Thorolfsdottir R.B., Jonsson B.A., Einarsson E., Gunnlaugsson G., Rognvaldsson S., Arnar D.O., Baldvinsson M., Bjarnason R.G. (2022). Multiomics study of nonalcoholic fatty liver disease. Nat. Genet..

[23] Wen H., Yang H.J., An Y.J., Kim J.M., Lee D.H., Jin X., Park S.W., Min K.J., Park S. (2013). Enhanced phase II detoxification contributes to beneficial effects of dietary restriction as revealed by multi-platform metabolomics studies. Mol. Cell. Proteom..

[24] Yang J., Trépo E., Nahon P., Cao Q., Moreno C., Letouzé E., Imbeaud S., Bayard Q., Gustot T., Deviere J. (2019). A 17-Beta-Hydroxysteroid Dehydrogenase 13 Variant Protects from Hepatocellular Carcinoma Development in Alcoholic Liver Disease. Hepatology.

[25] Tilson S.G., Morell C.M., Lenaerts A.S., Park S.B., Hu Z., Jenkins B., Koulman A., Liang T.J., Vallier L. (2021). Modeling *PNPLA3*-Associated NAFLD Using Human-Induced Pluripotent Stem Cells. Hepatology.

[26] Meroni M., Longo M., Fracanzani A.L., Dongiovanni P. (2020). MBOAT7 down-regulation by genetic and environmental factors predisposes to MAFLD. EBioMedicine.

[27] Ioannou G.N. (2021). Epidemiology and risk-stratification of NAFLD-associated HCC. J. Hepatol..

[28] Schneider C.V., Schneider K.M., Conlon D.M., Park J., Vujkovic M., Zandvakili I., Ko Y.A., Trautwein C., Carr R.M., Strnad P. (2021). A genome-first approach to mortality and metabolic phenotypes in *MTARC1* p.Ala165Thr (rs2642438) heterozygotes and homozygotes. Med.

[29] Thorn C.F., Aklillu E., Klein T.E., Altman R.B. (2012). PharmGKB summary: Very important pharmacogene information for CYP1A2. Pharmacogenet Genom..

[30] Dai D., Zeldin D.C., Blaisdell J.A., Chanas B., Coulter S.J., Ghanayem B.I., Goldstein J.A. (2001). Polymorphisms in human CYP2C8 decrease metabolism of the anticancer drug paclitaxel and arachidonic acid. Pharmacogenetics.

[31] Zi J., Liu D., Ma P., Huang H., Zhu J., Wei D., Yang J., Chen C. (2010). Effects of CYP2C9*3 and CYP2C9*13 on Diclofenac Metabolism and Inhibition-based Drug-Drug Interactions. Drug Metab. Pharmacokinet..

[32] Pharmgkb. https://www.pharmgkb.org/clinicalAnnotation/1450931522.

[33] Pharmgkb. https://www.pharmgkb.org/variantAnnotation/982044657.

[34] Yoon H., Shaw J.L., Haigis M.C., Greka A. (2021). Lipid metabolism in sickness and in health: Emerging regulators of lipotoxicity. Mol. Cell.

[35] Iyer K.R., Sinz M.W. (1999). Characterization of Phase I and Phase II hepatic drug metabolism activities in a panel of human liver preparations. Chem. Biol. Interact..

[36] Jancova P., Anzenbacher P., Anzenbacherova E. (2010). Phase II drug metabolizing enzymes. Biomed. Pap. Med. Fac. Univ. Palacky. Olomouc Czech Repub..

[37] Bessone F., Dirchwolf M., Rodil M.A., Razori M.V., Roma M.G. (2018). Review article: Drug-induced liver injury in the context of nonalcoholic fatty liver disease—A physiopathological and clinical integrated view. Aliment. Pharmacol. Ther..

[38] Danan G., Benichou C. (1993). Causality assessment of adverse reactions to drugs--I. A novel method based on the conclusions of international consensus meetings: Application to drug-induced liver injuries. J. Clin. Epidemiol..

[39] Danan G., Teschke R. (2015). RUCAM in Drug and Herb Induced Liver Injury: The Update. Int. J. Mol. Sci..

[40] Teschke R. (2018). Top-ranking drugs out of 3312 drug-induced liver injury cases evaluated by the Roussel Uclaf Causality Assessment Method. Expert. Opin. Drug Metab. Toxicol..

[41] Allard J., Le Guillou D., Begriche K., Fromenty B. (2019). Drug-induced liver injury in obesity and nonalcoholic fatty liver disease. Adv. Pharmacol..

[42] David S., Hamilton J.P. (2010). Drug-induced Liver Injury. US Gastroenterol. Hepatol. Rev..

[43] Fisher C.D., Lickteig A.J., Augustine L.M., Ranger-Moore J., Jackson J.P., Ferguson S.S., Cherrington N.J. (2009). Hepatic cytochrome P450 enzyme alterations in humans with progressive stages of nonalcoholic fatty liver disease. Drug Metab. Dispos..

[44] Benjamini Y., Hochberg Y. (1995). Controlling the False Discovery Rate: A Practical and Powerful Approach to Multiple Testing. J. R. Stat. Soc. Ser. B.

[45] Jager L.R., Leek J.T. (2014). An estimate of the science-wise false discovery rate and application to the top medical literature. Biostatistics.

[46] Cleves M.A. (2005). Exploratory Analysis of Single Nucleotide Polymorphism (SNP) for Quantitative Traits. Stata J..

