# Supplementary material for: Evaluation of Human Hepatocyte Drug Metabolism Carrying High-Risk or Protection-Associated Liver Disease Genetic Variants
Source: Int J Mol Sci. 2023 Aug 29;24(17):13406. doi: 10.3390/ijms241713406 (PMC10487897; doi:10.3390/ijms241713406)
Supplement: Supplementary file 1 [file ijms-24-13406-s001.zip › ijms-2503439-supplementary.pdf]

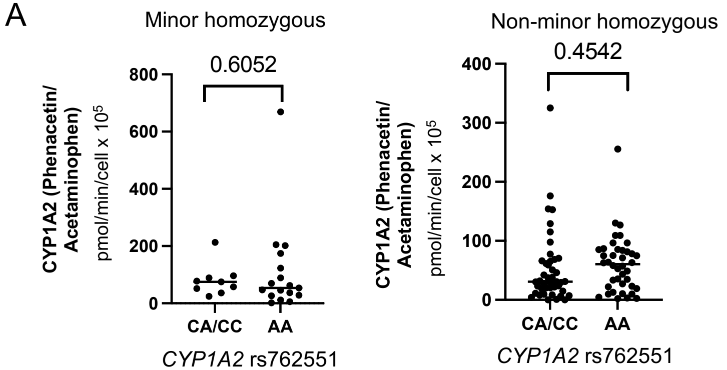

	MBOAT7 rs641738	
**CYP1A2 rs762551**	CC/CT	TT
CC/CA (number/%)	44/83,01%	9/16.98%
AA (number/%)	40/70.17%	17/29.82%

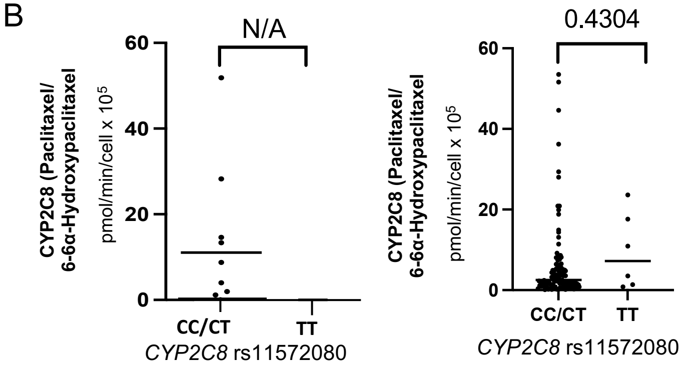

	PNPLA3 rs738409	
**CYP2C8 rs11572080**	CC/CG	GG
CC/CT (number/%)	91/91.91%	8/8.08%
TT (number/%)	6/100%	0/0%

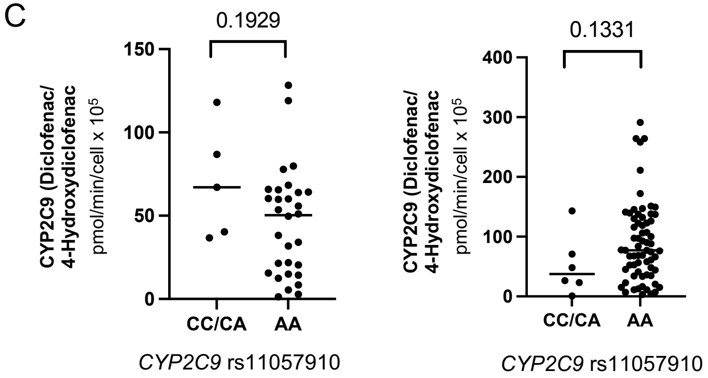

	HSD17B13 rs72613567	
**CYP2C9 rs1057910**	TT	TTA/TATA
CC/CA (number/%)	5/50%	5/50%
AA (number/%)	66/66%	34/34%

Supplementary Figure S1. Phase I CYP450 enzyme polymorphism genotyping among healthy donors. The box plots represent minor homozygous and non-minor homozygous genotypes for *CYP1A2* rs762551 in hepatocytes carrying *MBOAT7* rs641738 *p*=0.6052 and *p*=0.4542 (A), *CYP2C8* rs11572080 in hepatocytes carrying *PNPLA3* rs738409 *p*=not enough sample to evaluate and *p*=0.4304 (B), and *CYP2C9* rs1057910 in hepatocytes carrying *HSD17B13* rs72613567 *p*=0.1929 and *p*=0.1331 (C) in minor homozygous variant (right) and non-minor homozygous variant (left). The table on the left shows the number and percentage of hepatocytes that carry both minor and non-minor homozygous variants for *MBOAT7* rs641738, *PNPLA3* rs738409, *HSD17B13* rs72613567, and *CYP1A2, CYP2C8,* and *CYP2C9*.

Supplementary Table S1. Characteristics of primary donor hepatocytes

	PNPLA3 rs738409			MBOAT7 rs641738			GCKR rs780094			HSD17B13 rs72613567			MTARC rs2642438		
	CC	CG	GG	CC	CT	TT	CC	CT	TT	TT	TTA	TATA	GG	GA	AA
Healthy Liver Donor Individuals (n=126)															
**Characteristics**															
Age (mean yr ± SEM, n)	45.70 ± 2.05, 71	42.23 ± 2.69, 47	44.5 ± 3.84, 8	41.52 ± 3.18, 39	45.93 ± 2.04, 60	44.85 ± 3.28, 27	46.86 ± 2.42, 52	42.32 ± 2.27, 59	43.46 ± 4.39, 15	45.46 ± 1.83, 79	42.37 ± 2.81, 43	43 ± 14.61, 4	45.18 ± 2.12, 69	42.38 ± 2.58, 48	48.22 ± 4.31, 9
Gender (Female/Male)	28/43	29/18	6/2	20/19	28/32	15/12	27/25	27/32	9/6	40/39	21/22	2/2	36/33	22/26	5/4
Body-mass index (mean ± SEM, n)	29.87 ± 1.02, 69	28.80 ± 1.02, 46	32.07 ± 2.73, 8	28.60 ± 1.17, 37	30.29 ± 1.18, 59	29.51 ± 1.15, 27	29.44 ± 1.03, 51	29.37 ± 1.11, 57	31.12 ± 2.03, 15	30.05 ± 0.90, 78	28.83 ± 1.22, 42	29.29 ± 2.29, 3	29.07 ± 0.92, 67	29.86 ± 1.12, 47	32.35 ± 3.62, 9
Obesity (number, %)	28/41%	17/37%	4/50%	11, 30%	27, 46%	11, 41%	19, 37%	23, 40%	7, 47%	32, 41%	15, 36%	2, 67%	25, 37%	20, 43%	4, 44%
**Ethnicity**															
Caucasian (number)	56	30	4	25	44	21	37	45	8	53	36	1	43	39	8
Hispanic (number)	7	10	4	11	7	3	9	6	6	16	4	1	15	6	0
African-American (number)	7	3	0	2	6	2	5	5	0	9	1	0	9	1	0
Others or not provided (number)	2	3	0	2	2	1	1	3	1	1	2	2	2	2	1
**Total Population (%)**	56,35%	37,30%	6,35%	31,75%	46,9%	21,5%	41.27%	46,83%	11,90%	62,7%	34,13%	3,17%	54,8%	38,09%	7,14%

Supplementary Table S2: Linear regression test and Benjamini and Hochberg False Discovery Rate (FDR) method for Figure 1

CYP1A2 (Phenacetin/Acetaminophen)	Rank (i)	Variable	p-value	Benjamini-Hochberg critical value (i/m)Q	Notes
	1	MBOAT7_MH	0.011	0.014	SIGNIFICANT
	2	HSD17B13_MH	0.141	0.029	
	3	GCKR_MH	0.305	0.043	
	4	AGE	0.617	0.057	
	5	PNPLA3_MH	0.680	0.071	
	6	MTARC_MH	0.800	0.086	
	7	GENDER	0.929	0.100	
CYP2C8 (Paclitaxel/6-6α-Hydroxypaclitaxel)	Rank (i)	Variable	p-value	Benjamini-Hochberg critical value (i/m)Q	Notes
	1	PNPLA3 MH	0.004	0.014	SIGNIFICANT
	2	MBOAT7_MH	0.084	0.029	
	3	HSD17B13_MH	0.106	0.043	
	4	GCKR_MH	0.243	0.057	
	5	MALE	0.320	0.071	
	6	MTARC_MH	0.401	0.086	
	7	GENDER	0.576	0.100	
CYP2C9 (Diclofenac/4-Hydroxydiclofenac)	Rank (i)	Variable	p-value	Benjamini-Hochberg critical value (i/m)Q	Notes
	1	HSD17B13_MH	0.001	0.014	SIGNIFICANT
	2	GENDER	0.140	0.029	
	3	AGE	0.156	0.043	
	4	MTARC_MH	0.274	0.057	
	5	MBOAT7_MH	0.747	0.071	
	6	PNPLA3_MH	0.838	0.086	
	7	GCKR_MH	0.992	0.100	
CYP2D6 (Dextromethorphan/Dextrorphan)	Rank (i)	Variable	p-value	Benjamini-Hochberg critical value (i/m)Q	Notes
	1	MBOAT7_MH	0.277	0.014	
	2	GCKR_MH	0.452	0.029	
	3	AGE	0.546	0.043	
	4	HSD17B13_MH	0.621	0.057	
	5	GENDER	0.634	0.071	
	6	PNPLA3 MH	0.822	0.086	
	7	MTARC_MH	0.824	0.100	
CYP2E1 (Chlorzoxazone/6-Hydroxychlorzoxazone)	Rank (i)	Variable	p-value	Benjamini-Hochberg critical value (i/m)Q	Notes
	1	MBOAT7_MH	0.194	0.014	
	2	GCKR_MH	0.423	0.029	
	3	GENDER	0.464	0.043	
	4	PNPLA3 MH	0.482	0.057	
	5	HSD17B13_MH	0.801	0.071	
	6	MTARC_MH	0.861	0.086	
	7	AGE	0.942	0.100	
CYP3A4 (Midazolam/1-Hydroxymidazolam	Rank (i)	Variable	p-value	Benjamini-Hochberg critical value (i/m)Q	Notes
	1	GENDER	0.279	0.014	
	2	MBOAT7_MH	0.342	0.029	
	3	GCKR_MH	0.674	0.043	
	4	AGE	0.761	0.057	
	5	HSD17B13_MH	0.814	0.071	
	6	PNPLA3_MH	0.900	0.086	
	7	MTARC_MH	0.971	0.100	

Supplementary Table S3: Linear regression test and Benjamini and Hochberg False Discovery Rate (FDR) method for Figure 2

ECOD (7-Ethoxycoumarin/7-OH Coumarin Glucuronide)	Rank (i)	Variable	p-value	Benjamini-Hochberg critical value (i/m)Q	Notes
	1	GENDER	0.142	0.014	
	2	MBOAT7_MH	0.189	0.029	
	3	MTARC_MH	0.345	0.043	
	4	AGE	0.399	0.057	
	5	HSD17B13_MH	0.559	0.071	
	6	GCKR_MH	0.746	0.086	
	7	PNPLA3_MH	0.828	0.100	
**ECOD (7-Ethoxycoumarin/7-OH Coumarin)**	**Rank (i)**	**Variable**	**p-value**	**Benjamini-Hochberg critical value (i/m)Q**	**Notes**
	1	PNPLA3_MH	0.102	0.014	
	2	GENDER	0.401	0.029	
	3	AGE	0.499	0.043	
	4	MBOAT7_MH	0.501	0.057	
	5	MTARC_MH	0.508	0.071	
	6	HSD17B13_MH	0.717	0.086	
	7	GCKR_MH	0.866	0.100	
**FMO (Benzydamine HCl/Benzydamine-N-Oxide)**	**Rank (i)**	**Variable**	**p-value**	**Benjamini-Hochberg critical value (i/m)Q**	**Notes**
	1	AGE	0.003	0.014	SIGNIFICANT
	2	GENDER	0.138	0.029	
	3	HSD17B13_MH	0.276	0.043	
	4	MBOAT7_MH	0.474	0.057	
	5	GCKR_MH	0.739	0.071	
	6	MTARC_MH	0.874	0.086	
	7	PNPLA3_MH	0.992	0.100	
**MAO (Kynuramine HCl/4-hydroxyquinoline)**	**Rank (i)**	**Variable**	**p-value**	**Benjamini-Hochberg critical value (i/m)Q**	**Notes**
	1	AGE	0.109	0.014	
	2	MBOAT7_MH	0.306	0.029	
	3	MTARC_MH	0.421	0.043	
	4	GENDER	0.755	0.057	
	5	PNPLA3_MH	0.884	0.071	
	6	HSD17B13_MH	0.940	0.086	
	7	GCKR_MH	0.965	0.100	
**AO (Carbezeran HCl/4-Hydroxycarbazeran)**	**Rank (i)**	**Variable**	**p-value**	**Benjamini-Hochberg critical value (i/m)Q**	**Notes**
	1	AGE	0.023	0.014	
	2	MTARC_MH	0.187	0.029	
	3	MBOAT7_MH	0.406	0.043	
	4	HSD17B13_MH	0.662	0.057	
	5	GCKR_MH	0.837	0.071	
	6	GENDER	0.868	0.086	
	7	PNPLA3_MH	0.974	0.100	
**CES2 (Irinotecan/SN38)**	**Rank (i)**	**Variable**	**p-value**	**Benjamini-Hochberg critical value (i/m)Q**	**Notes**
	1	HSD17B13_MH	0.114	0.014	
	2	PNPLA3_MH	0.210	0.029	
	3	MBOAT7_MH	0.573	0.043	
	4	GENDER	0.615	0.057	
	5	MTARC_MH	0.675	0.071	
	6	GCKR_MH	0.798	0.086	
	7	AGE	0.922	0.100	

Supplementary Table S4: Linear regression test and Benjamini and Hochberg False Discovery Rate (FDR) method for Figure 3

SULT (7-Ethoxycoumarin/7-OH Coumarin Sulfate)	Rank (i)	Variable	p-value	Benjamini-Hochberg critical value (i/m)Q	Notes
	1	HSD17B13_MH	0.093	0.014	
	2	MTARC_MH	0.099	0.029	
	3	GENDER	0.099	0.043	
	4	GCKR_MH	0.299	0.057	
	5	MBOAT7_MH	0.517	0.071	
	6	PNPLA3_MH	0.572	0.086	
	7	AGE	0.848	0.100	

SULT (Acetaminophen/Acetaminophen Sulfate)	Rank (i)	Variable	p-value	Benjamini-Hochberg critical value (i/m)Q	Notes
	1	AGE	0.028	0.014	
	2	GENDER	0.032	0.029	
	3	GCKR_MH	0.292	0.043	
	4	MBOAT7_MH	0.303	0.057	
	5	MTARC_MH	0.555	0.071	
	6	PNPLA3_MH	0.708	0.086	
	7	HSD17B13_MH	0.870	0.100	

UGT (7-Ethoxycoumarin/7-OH Coumarin Glucuronide	Rank (i)	Variable	p-value	Benjamini-Hochberg critical value (i/m)Q	Notes
	1	GCKR_MH	0.094	0.014	
	2	MBOAT7_MH	0.190	0.029	
	3	MTARC_MH	0.317	0.043	
	4	HSD17B13_MH	0.571	0.057	
	5	GENDER	0.666	0.071	
	6	AGE	0.761	0.086	
	7	PNPLA3_MH	0.963	0.100	

UGT (Acetaminophen/Acetaminophen Glucuronide)	Rank (i)	Variable	p-value	Benjamini-Hochberg critical value (i/m)Q	Notes
	1	GCKR_MH	0.068	0.014	
	2	MBOAT7_MH	0.377	0.029	
	3	AGE	0.506	0.043	
	4	HSD17B13_MH	0.535	0.057	
	5	PNPLA3_MH	0.654	0.071	
	6	GENDER	0.690	0.086	
	7	MTARC_MH	0.945	0.100	

GST (Acetaminophen/Acetaminophen Glutathione)	Rank (i)	Variable	p-value	Benjamini-Hochberg critical value (i/m)Q	Notes
	1	GENDER	0.137	0.014	
	2	AGE	0.207	0.029	
	3	GCKR_MH	0.270	0.043	
	4	MBOAT7_MH	0.455	0.057	
	5	HSD17B13_MH	0.661	0.071	
	6	MTARC_MH	0.731	0.086	
	7	PNPLA3_MH	0.785	0.100	

NAT1 (4-Aminobenzoic HCl/N-Acetyl-p-a)	Rank (i)	Variable	p-value	Benjamini-Hochberg critical value (i/m)Q	Notes
	1	GENDER	0.086	0.014	
	2	GCKR_MH	0.137	0.029	
	3	AGE	0.179	0.043	
	4	HSD17B13_MH	0.294	0.057	
	5	MTARC_MH	0.612	0.071	
	6	PNPLA3_MH	0.665	0.086	
	7	MBOAT7_MH	0.816	0.100	

NAT2 (Sulfamethazine/N-Acetyl-sulfamethazine)	Rank (i)	Variable	p-value	Benjamini-Hochberg critical value (i/m)Q	Notes
	1	HSD17B13_MH	0.025	0.014	
	2	MBOAT7_MH	0.093	0.029	
	3	MTARC_MH	0.107	0.043	
	4	PNPLA3_MH	0.173	0.057	
	5	AGE	0.320	0.071	
	6	GENDER	0.388	0.086	
	7	GCKR_MH	0.393	0.100	

Supplementary Table S5: Linear regression test and Benjamini and Hochberg False Discovery Rate (FDR) method for Figure 4

CYP1A2 (Omeprazole/Phenacetin)	Rank (i)	Variable	p-value	Benjamini-Hochberg critical value (i/m)Q	Notes
	1	PNPLA3_MH	0.015	0.014	SIGNIFICANT
	2	MBOAT7_MH	0.017	0.029	SIGNIFICANT
	3	MTARC_MH	0.094	0.043	
	4	GENDER	0.370	0.057	
	5	GCKR_MH	0.397	0.071	
	6	HSD17B13_MH	0.524	0.086	
	7	AGE	0.858	0.100	
CYP2B6(Phenobarbital/Bupropion)	Rank (i)	Variable	p-value	Benjamini-Hochberg critical value (i/m)Q	Notes
	1	AGE	0.039	0.014	
	2	MTARC_MH	0.117	0.029	
	3	PNPLA3_MH	0.257	0.043	
	4	MBOAT7_MH	0.275	0.057	
	5	HSD17B13_MH	0.284	0.071	
	6	GENDER	0.569	0.086	
	7	GCKR_MH	0.600	0.100	
CYP3A4(Rifampin/Testosterone)	Rank (i)	Variable	p-value	Benjamini-Hochberg critical value (i/m)Q	Notes
	1	GCKR_MH	0.133	0.014	
	2	MBOAT7_MH	0.215	0.029	
	3	PNPLA3_MH	0.493	0.043	
	4	HSD17B13_MH	0.604	0.057	
	5	AGE	0.625	0.071	
	6	MTARC_MH	0.711	0.086	
	7	GENDER	0.989	0.100	

Supplementary Table S6: Linear regression test and Benjamini and Hochberg False Discovery Rate (FDR) method for Figure 5

CYP1A2 (Omeprazole/Phenacetin)	Rank (i)	Variable	p-value	Benjamini-Hochberg critical value (i/m)Q	Notes
	1	AGE	0.211	0.014	
	2	HSD17B13_MH	0.249	0.029	
	3	GCKR_MH	0.297	0.043	
	4	MBOAT7_MH	0.361	0.057	
	5	GENDER	0.462	0.071	
	6	MTARC_MH	0.703	0.086	
	7	PNPLA3_MH	0.999	0.100	
**CYP2B6 (Phenobarbital /Bupropion)**	**Rank (i)**	**Variable**	**p-value**	**Benjamini-Hochberg critical value (i/m)Q**	**Notes**
	1	AGE	0.000	0.014	
	2	HSD17B13_MH	0.271	0.029	
	3	MBOAT7_MH	0.450	0.043	
	4	MTARC_MH	0.488	0.057	
	5	PNPLA3_MH	0.605	0.071	
	6	GCKR_MH	0.696	0.086	
	7	GENDER	0.868	0.100	
**CYP2C8 (Rifampin/Paclitaxel)**	**Rank (i)**	**Variable**	**p-value**	**Benjamini-Hochberg critical value (i/m)Q**	**Notes**
	1	GENDER	0.103	0.014	
	2	AGE	0.105	0.029	
	3	MBOAT7_MH	0.481	0.043	
	4	HSD17B13_MH	0.534	0.057	
	5	MTARC_MH	0.642	0.071	
	6	PNPLA3_MH	0.782	0.086	
	7	GCKR_MH	0.913	0.100	
**CYP2C9 (Rifampin/Diclofenac)**	**Rank (i)**	**Variable**	**p-value**	**Benjamini-Hochberg critical value (i/m)Q**	**Notes**
	1	MTARC_MH	0.266	0.014	
	2	GENDER	0.344	0.029	
	3	HSD17B13_MH	0.804	0.043	
	4	MBOAT7_MH	0.850	0.057	
	5	AGE	0.857	0.071	
	6	PNPLA3_MH	0.898	0.086	
	7	GCKR_MH	0.925	0.100	
**CYP2C19 (Rifampin/S-mephenytoin)**	**Rank (i)**	**Variable**	**p-value**	**Benjamini-Hochberg critical value (i/m)Q**	**Notes**
	1	AGE	0.014	0.014	
	2	PNPLA3_MH	0.120	0.029	
	3	GCKR_MH	0.130	0.043	
	4	MTARC_MH	0.162	0.057	
	5	MBOAT7_MH	0.278	0.071	
	6	GENDER	0.440	0.086	
	7	HSD17B13_MH	0.981	0.100	
**CYP3A4 (Rifampin/Testosterone)**	**Rank (i)**	**Variable**	**p-value**	**Benjamini-Hochberg critical value (i/m)Q**	**Notes**
	1	MBOAT7_MH	0.076	0.014	
	2	HSD17B13_MH	0.119	0.029	
	3	GCKR_MH	0.339	0.043	
	4	AGE	0.478	0.057	
	5	GENDER	0.599	0.071	
	6	MTARC_MH	0.917	0.086	
	7	PNPLA3_MH	0.993	0.100	

Supplementary Table S7. Gene variants IDs used for genotyping.

**GENOTYPING**		
**Target Gene**	**Company**	**Gene Expression Assay ID / Catalogue No.**
PNPLA3 rs738409	ThermoFisher Scientific	C_7241_10 / 4351376
MBOAT7 rs641738	ThermoFisher Scientific	C_8716820_10 / 4351376
GCKR rs780094	ThermoFisher Scientific	C_2862873_10 / 4351376
HSD17B13 rs72613567	ThermoFisher Scientific	ANGZVM6 [1] / 4331349
MTARC1 rs2642438	ThermoFisher Scientific	C_1235772_10 / 4351376
CYP2C8 rs11572080	ThermoFisher Scientific	C__25625794_60/ 4362691
CYP2C9 rs1057910	ThermoFisher Scientific	C__27104892_10/4362691
CYP1A2 rs762551	ThermoFisher Scientific	C___8881221_40/4362691
